# Metabolic Syndrome Is Not Associated With Prostate Cancer Recurrence: A Retrospective Analysis of a Chinese Cohort

**DOI:** 10.3389/fonc.2020.00063

**Published:** 2020-01-30

**Authors:** Xin Xu, Qinchen Li, Chengdong Chang, Xiao Wang, Liping Xie

**Affiliations:** ^1^Department of Urology, School of Medicine, The First Affiliated Hospital, Zhejiang University, Hangzhou, China; ^2^Department of Pathology, School of Medicine, The First Affiliated Hospital, Zhejiang University, Hangzhou, China

**Keywords:** prostate cancer, metabolic syndrome, radical prostatectomy, biochemical recurrence, cohort

## Abstract

**Objective:** Metabolic syndrome (MetS), a common disease that affects many people around the world, has been hypothesized to be associated with human cancers, including prostate cancer (PCa), but the association has not been consistent. The aim of the current study was to evaluate whether MetS and its components are risk factors for PCa biochemical recurrence (BCR) among a cohort of postoperative patients at our hospital in China.

**Materials and Methods:** This retrospective study included 214 patients with PCa who received radical prostatectomy. Differences between groups were estimated using the χ^2^ test or Student's *t-*test. BCR rates were calculated according to the Kaplan-Meier method with the log-rank test. A Cox regression analysis was conducted for the multivariate analyses to identify significant predictors of BCR.

**Results:** Of the 214 eligible men, 55 experienced BCR and 24 met the MetS diagnostic criteria. Multivariate Cox model analysis showed that patients with BCR had a higher Gleason score [hazard ratio (HR) 2.51, 95% confidence interval (CI) 1.33–4.76] and positive nerve invasion (HR 3.57, 95% CI 1.85–6.88). MetS was not associated with BCR (HR 0.38, 95% CI 0.13–1.10).

**Conclusion:** BCR is not associated with MetS but is associated with a higher Gleason score and positive nerve invasion.

## Introduction

Prostate cancer (PCa) has been rated as the most commonly diagnosed cancer in men in the United States according to a report published in 2019 (1). With the changes in lifestyle in recent years, the incidence rate of PCa in China has increased, and there has been an upward trend in age-standardized mortality rates observed for PCa in men (2). Moreover, adverse lifestyle factors, such as alcohol consumption, smoking, overnutrition, sedentary lifestyle, obesity, western-type diets, and hypertension-inducing diets, could accelerate the onset of metabolic syndrome (MetS) (3).

Metabolic syndrome (MetS), including central obesity, raised blood pressure, elevated fasting glucose and dyslipidemia (low high-density lipoprotein cholesterol and elevated triglycerides), is a cluster of metabolic abnormalities related to insulin resistance (4). MetS and its components have become more prevailing global public health issues (5), and growing evidence has established an association between MetS and diseases, including cirrhosis (6), non-alcoholic fatty liver disease (3), diabetes mellitus (3), and cardiovascular disease (7). Moreover, emerging evidence indicates that MetS and its components were associated with an increased risk of PCa biochemical recurrence (BCR) (8, 9), which was defined as being present in the event of two consecutive increasing postoperative serum prostate-specific antigen (PSA) values ≥0.2 ng/ml among prostate cancer patients who underwent radical prostatectomy (RP). In addition, several studies have shown that MetS or its components were related to adverse clinical and pathological characteristics in individuals with PCa after RP, involving a positive surgical margin (8, 10, 11), extracapsular extension (8), pathological Gleason score (GS) (8, 10), PCa-specific mortality and local recurrence (10) and PCa biochemical recurrence (12, 13). However, different conclusions provided by another study revealed no association between MetS and PCa risk in the overall analyses (14), and PCa detection risk (15), and PCa biochemical recurrence (10–12). Clearly, inconsistent results regarding MetS and BCR still exist.

Given the unclear association between PCa BCR and MetS and the increasing prevalence of MetS, we hypothesized that MetS or its components might be associated with an increased risk for PCa BCR. Therefore, in the present study, we investigated a range of preoperative MetS components, including body mass index (BMI), blood pressure, fasting glucose, high-density lipoprotein cholesterol (HDL) and triglycerides (TGs), as well as the preoperative serum PSA levels of patients with clinically localized PCa who received RP, particularly with respect to the link between these indicators and BCR after surgery. The aim of the current study was to evaluate whether MetS and its components are risk factors for PCa BCR among a cohort of postoperative patients at our hospital in China to prevent increased prostate cancer mortality associated with MetS.

## Patients and Methods

### Study Subjects

Men with clinically localized PCa undergoing radical prostatectomy at the Department of Urology at the First Affiliated Hospital of Zhejiang University from January 2013 to December 2015 were, respectively, included in this study. Exclusion criteria: patients who received neoadjuvant hormonal therapy; components of MetS were not completed; PSA levels did not reach a nadir ≤0.2 ng/mL after surgery; loss to follow-up. A total of 214 patients were finally included in our study. Clinicopathological information, including age, height, weight, history of hypertension and diabetes, lipid profiles, preoperative prostate—specific antigen (PSA) levels, prostatectomy GS, and pathological characteristics, was gathered from medical records and analyzed. BCR was defined as two consecutive rising detectable PSA concentrations of >0.2 ng/mL or the administration of adjuvant therapy during the postoperative follow-up period. Higher GS was defined as GS ≥8. The study protocol was approved by the Institutional Research Review Boards of the First Affiliated Hospital of Zhejiang University. Due to the retrospective nature of the study, informed consent was waived. All methods were performed in accordance with the relevant guidelines and regulations.

### Metabolic Syndrome Definition

MetS was defined according to the criteria recommended by the Chinese Diabetes Society (16), which requires that at least three of four of the following standards are met: (1) overweight and obesity: body mass index (BMI) ≥25 kg/m2; (2) hypertension: systolic and diastolic blood pressure ≥140 and 90 mmHg, respectively, on three consecutive occasions, or a physician's diagnosis of hypertension; (3) diabetes: fasting glucose ≥6.1 mmol/l, or a physician's diagnosis; (4) dyslipidemia (hypertriglyceridemia and/or low high-density lipoprotein (HDL) level): serum triglyceride (TG) level ≥1.7 mmol/l and/or HDL-cholesterol level <0.9 mmol/l.

### Statistical Analyses

Differences between groups were estimated using the χ2 test or Student's *t*-test. BCR rates were calculated according to the Kaplan-Meier method with log-rank test. A Cox regression analysis (proportional hazards model) was conducted for the multivariate analyses to identify significant predictors of BCR. All analyses were performed using SPSS16.0 software (IBM, USA) and a two-tailed value of *P* < 0.05 was considered to be statistically significant.

## Results

Table 1 shows the main characteristics of patients. A total 214 patients met the inclusion criteria for the study, 55 experienced BCR and 24 met diagnosis criteria of MetS. The mean (SD) of age was 65.9 (6.48) years, the mean (SD) of serum PSA level was 14.1 (9.00) ng/mL, and the BMI (SD) was 23.8 (2.67) m^2^/kg. The patients with MetS showed no difference in the age and the level of PSA when compared to the Non-MetS group (*P* = 0.16 and *P* = 0.67, respectively) as shown in Table 2. Similarly, when compared MetS with Non-MetS group, no significant difference was observed for various clinicopathological PCa features, including prostate capsule invasion, seminal vesicle invasion, nerve invasion, positive surgical margin, lymph node involvement and prostatectomy GS (Table 2, all *P* > 0.05).

**Table 1:** Patient characteristics (*n* = 214).

**Variables**	**Values**
Age, years	65.92 ± 6.48
BMI	23.83 ± 2.67
Total PSA, ng/ml	14.07 ± 9.00
**Smoke status**	
Yes	106 (49.5%)
No	108 (50.5%)
**Diabetes**	
Yes	28 (13.1%)
No	186 (86.9%)
**Hypertension**	
Yes	108 (50.5%)
No	106 (49.5%)
**Prostate capsule invasion**	
Yes	76 (35.5%)
No	138 (64.5%)
**Seminal vesicle invasion**	
Yes	25 (11.7%)
No	189 (88.3%)
**Nerve invasion**	
Yes	79 (36.9%)
No	135 (63.1%)
**Surgical margin**	
Yes	56 (26.2%)
No	158 (73.8%)
**Nodal status**	
Yes	4 (1.9%)
No	210 (98.1%)
**Gleason score**	
6	35 (16.4%)
7	137 (64.0%)
8	20 (9.3%)
9	22 (10.3%)

*BMI, body mass index; PSA, prostate-specific antigen*.

**Table 2:** Association between MetS and clinicopathological factors.

**Variables**	**Non-MetS (*n* = 190)** **(88.79%)**	**MetS (*n* = 24)** **(11.21%)**	***P*-value**
Age, years	65.70 ± 6.53	67.67 ± 5.90	0.16
Total PSA, ng/ml	14.17 ± 9.12	13.33 ± 8.07	0.67
**Smoke status**			
Yes	96 (50.5%)	10 (41.7%)	0.41
No	94 (49.5%)	14 (58.3%)	
**Prostate capsule invasion**			
Yes	70 (36.8%)	6 (25.0%)	0.25
No	120 (63.2%)	18 (75.0%)	
**Seminal vesicle invasion**			
Yes	23 (12.1%)	2 (8.3%)	0.59
No	167 (87.9%)	22 (91.7%)	
**Nerve invasion**			
Yes	71 (37.4%)	8 (33.3%)	0.70
No	119 (62.6%)	16 (66.7%)	
**Positive surgical margin**			
Yes	50 (26.3%)	6 (25.0%)	0.89
No	140 (73.7%)	18 (75.0%)	
**Lymph node involvement**			
Yes	4 (2.1%)	0 (0.0%)	0.47
No	186 (97.9%)	24 (100%)	
**Prostatectomy GS**			
≥8	36 (18.9%)	6 (25.0%)	0.48
<8	154 (81.1%)	18 (75.0%)	

*MetS, Metabolic syndrome; PSA, prostate-specific antigen*.

Table 3 indicates 55 (25.7%) patients underwent BCR after RP and 159 (74.30%) patients was not. Assessment of differences in clinicopathological characteristics with chi-squared tests associated with BCR revealed that some variables were significantly associated with BCR including: prostate capsule invasion, seminal vesicle invasion, nerve invasion and prostatectomy GS (*P* < 0.01, *P* = 0.03, *P* < 0.01, and *P* < 0.01, respectively). Unexpectedly, Table 4 exhibited that MetS and its components were rarely significant associated with BCR when compared BCR with BCR-free group (all *P* > 0.05).

**Table 3:** Association between BCR and clinicopathological factors.

**Variables**	**BCR-free group (*n* = 159)** **(74.30%)**	**BCR group (*n* = 55)** **(25.70%)**	***P*-value**
Age, years	65.95 ± 6.77	65.84 ± 5.60	0.91
Total PSA, ng/ml			
**Smoke status**			
Yes	80 (50.3%)	26 (47.3%)	0.70
No	79 (49.7%)	29 (52.7%)	
**Prostate capsule invasion**			
Yes	44 (27.7%)	32 (58.2%)	**<0.01**
No	115 (72.3%)	23 (41.8%)	
**Seminal vesicle invasion**			
Yes	14 (8.8%)	11 (20.0%)	**0.03**
No	145 (91.2%)	44 (80.0%)	
**Nerve invasion**			
Yes	41 (25.8%)	38 (69.1%)	**<0.01**
No	118 (74.2%)	17 (30.9%)	
**Positive surgical margin**			
Yes	40 (25.2%)	16 (29.1%)	0.57
No	119 (74.8%)	39 (70.9%)	
**Lymph node involvement**			
Yes	2 (1.3%)	2 (3.6%)	0.26
No	157 (98.7%)	53 (96.4%)	
**Prostatectomy GS**			
≥8	19 (11.9%)	23 (41.8%)	**<0.01**
<8	140 (88.1%)	32 (58.2%)	

*BCR, biochemical recurrence; PSA, prostate-specific antigen; GS, Gleason score*.

*Bold value indicates statistically significant*.

**Table 4:** Association between BCR and MetS.

**Variables**	**BCR-free group (*n* = 159)**	**BCR group (*n* = 55)**	***P*-value**
**MetS**
Yes	20 (12.6%)	4 (7.3%)	0.28
No	139 (87.4%)	51 (92.7%)	
**Hypertension**			
Yes	81 (50.9%)	27 (49.1%)	0.81
No	78 (49.1%)	28 (50.9%)	
**Diabetes**			
Yes	22 (13.8%)	6 (10.9%)	0.58
No	137 (86.2%)	49 (89.1%)	
**BMI**			
<25	109 (68.6%)	38 (69.1%)	0.94
≥25	50 (31.4%)	17 (30.9%)	
**Hypertriglyceridemia**			
Yes	35 (22.0%)	10 (18.2%)	0.55
No	124 (78.0%)	45 (81.8%)	
**Low HDL-cholesterol**			
Yes	32 (20.1%)	10 (18.2%)	0.75
No	127 (79.9%)	45 (81.8%)	

*BCR, biochemical recurrence; MetS, Metabolic syndrome; HDL, high-density lipoprotein; BMI, body mass index; HDL, high-density lipoprotein cholesterol*.

**Figure 1:**
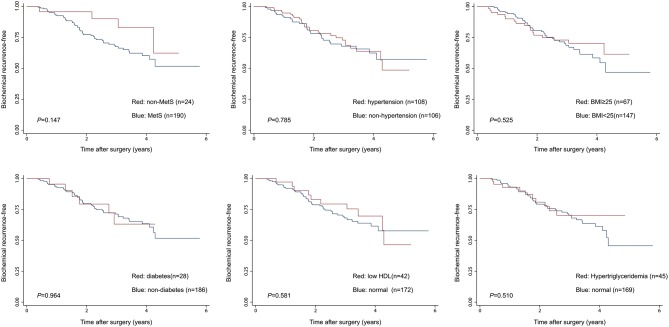
The Kaplan-Meier analysis for BCR-free survival according to the presence of MetS and its components. The log-rank test was performed to determine the statistical significance between the two groups.

**Figure 2:**
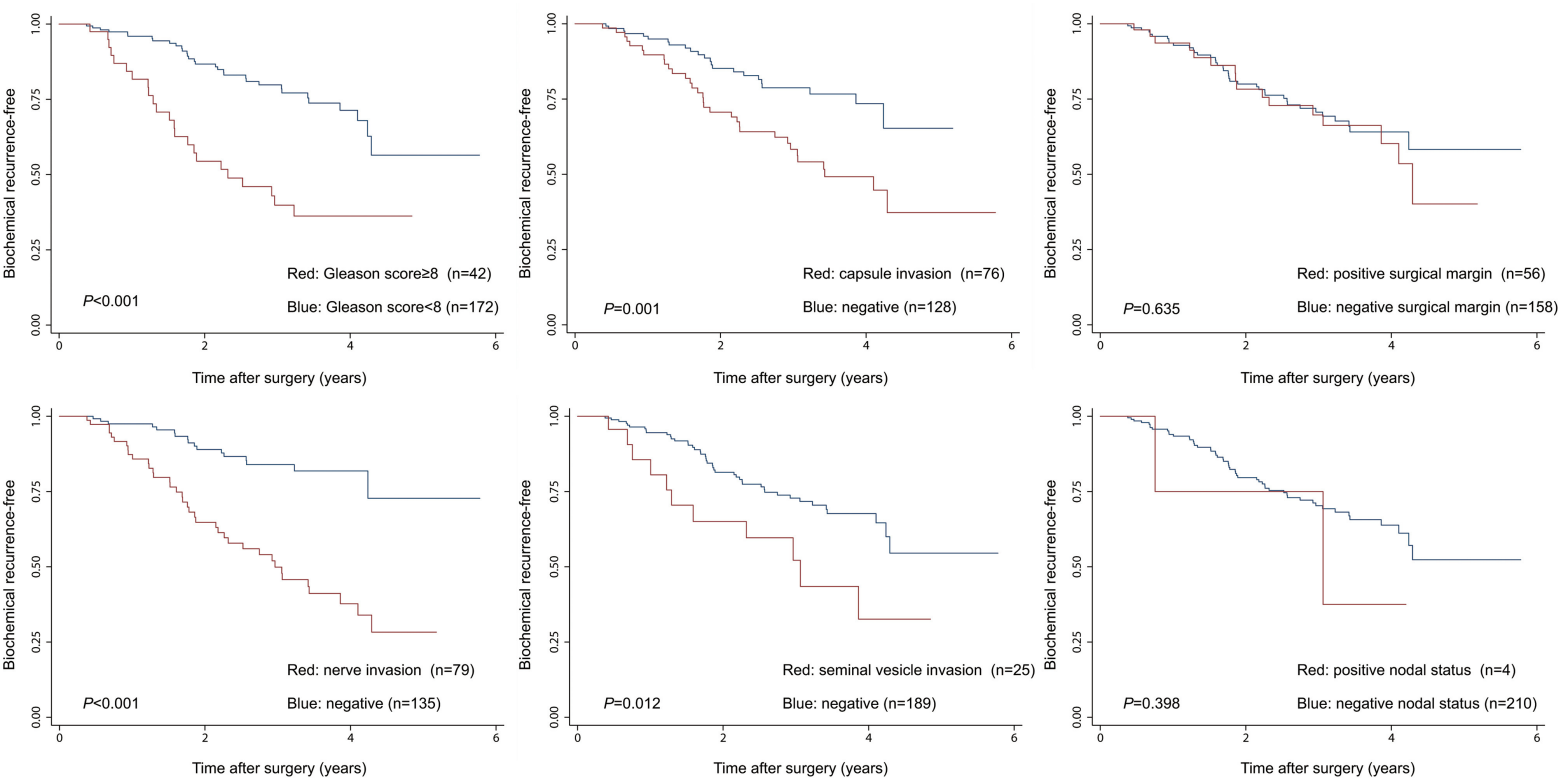
The Kaplan-Meier analysis for BCR-free survival according to clinicopathological PCa features. The log-rank test was performed to determine the statistical significance between the two groups.

Given the above results, we take Kaplan–Meier curves to further demonstrated that BCR was associated with MetS (*P* = 0.147), hypertension (*P* = 0.785), BMI (*P* = 0.525), diabetes (*P* = 0.964), low (HDL) (*P* = 0.581) and hypertriglyceridemia level (*P* = 0.510) (Figure 1). To better select which patients with BCR had worse survival outcomes, all patients were further stratified according to clinicopathological PCa features, including prostate capsule invasion, seminal vesicle invasion, nerve invasion, positive surgical margin, lymph node involvement and prostatectomy GS, the results are shown in Figure 2. Patients with BCR had increased GS (*P* < 0.001), positive prostate capsule invasion (*P* = 0.001), positive nerve invasion (*P* < 0.001) and positive seminal vesicle invasion (*P* = 0.012). However, in the multivariate Cox model analysis (Table 5), only the presence of nerve invasion (HR 3.57, 95% CI 1.85–6.88) and advanced pathologic GS (HR 2.51, 95% CI 1.33–4.76) were identified as significant BCR predictors.

**Table 5:** Multivariate Cox model analysis of BCR.

**Variables**	**HR (95% CI)**	***P*-value**
MetS (yes vs. no)	0.38 (0.13–1.10)	0.074
Age (continuous)	1.02 (0.98–1.07)	0.322
Total PSA (continuous)	1.01 (0.99–1.04)	0.344
Smoking status (yes vs. no)	0.82 (0.47–1.42)	0.481
Prostate capsule invasion (yes vs. no)	1.58 (0.86–2.90)	0.143
Seminal vesicle invasion (yes vs. no)	1.09 (0.50–2.40)	0.826
Nerve invasion (yes vs. no)	**3.57 (1.85–6.88)**	**<0.001**
Positive surgical margin (yes vs. no)	0.53 (0.28–1.02)	0.057
Positive nodal status (yes vs. no)	1.63 (0.34–7.74)	0.542
Gleason score (≥8 vs. <8)	**2.51 (1.33–4.76)**	**0.005**

*BCR, biochemical recurrence; HR, hazard ratio; CI, confidence interval; MetS, Metabolic syndrome; PSA, prostate-specific antigen. Bold value indicates statistically significant*.

## Discussion

Prostate cancer is the most common carcinoma in men with nearly 174,650 new cases annually in US (1). Radical prostatectomy (RP) or radiation therapy (RT) is the most prevailing primary treatments for patients of PCa, which generally provided excellent cancer control. Unfortunately, PCa recurrence following primary therapy generally happened, with the incidence of BCR around 35% at 10 years following RP (17). Conventionally, high-risk patients of PCa had an increased risk of BCR, including following features, rapidly rising PSA, Gleason score 8–10, and so on. Many studies recently focused on the relationships between BCR and MetS, and had reported inconsistent results. Therefore, we performed a comprehensive analysis for patients with PCa about the associations between BCR and MetS in the current study. The results in this study showed there was no significant correlation between BCR and MetS as well as its components among the cohort of postoperative patients of our hospital in China. BCR was only associated with increased GS and positive nerve invasion in multivariate analysis.

In our present report, patients of PCa with MetS didn't have a higher risk of BCR, and pathologic characteristics also didn't differ between patients according to whether they had MetS. But previous studies have linked MetS to an increased risk of higher GS, lymph node involvement and pT3–4 diseases (18) or some MetS components increase risk PCa recurrence (13). According to a meta-analysis, showing a consistent conclusion, it indicated a higher likelihood of advanced, high-grade or BCR of PCa among those with MetS (14). In contrast to above findings, Macleod et al. found that PCa recurrence was not associated with MetS [multivariable: HR 0.96, 95% CI (0.61–1.50); propensity adjusted HR 1.04, 95% CI (0.67–1.62)] (12), which showing no difference in our results [HR 0.38, 95% CI (0.13–1.10), *P* = 0.074].

Taking a further analysis, our results showed any components related with MetS didn't increase the risk of BCR, which was in accordance with existing research. For instance, Dickerman et al. reported that obesity (BMI) and weight change were not associated with an increased risk of BCR, weight gain might early influence prostatic carcinogenesis and might play a part in the development of cancers more likely to progress (19). A previous meta-analysis revealed that diabetes mellitus was obviously negatively related with prostate cancer risk in population-based studies (RR = 0.72, 95% CI: 0.64-0.81) (20). And existing evidence exhibited patients with hypertriglyceridemia had a better BCR-free survival rate than those not, meaning that preoperative hypertriglyceridemia was related with a lower risk of BCR in patients with clinically localized PCa (21). Although Macleod et al. did not confirm the association between BCR and hypertension, the further investigation of the role for hypertension was deserved (12). Second hypertension is related to age. Although several studies have showed that hypertension is associated with increased prostate cancer incidence. Finally, smoking is also a factor that possibly has an important role. Several studies have indicated that smoking is potentially associated with more aggressive prostate cancer and 50% of the patients in our study are smokers.

The natural history of BCR after RP could be long but variable. Better risk assessment models were merited to recognize patients who were at high risk for prostate cancer-specific death early and may benefit from aggressive salvage treatment and to realize patients who were at greater odds for prostate cancer-specific survival and could be safely observed. Existing data in our study signed that BCR was associated with adverse clinicopathological features, including increased GS and positive nerve invasion. It meant patients with adverse clinicopathological features was associated with a more aggressive tumor phenotype, could be regarded as the poor prognosis indicators for BCR in patients with PCa and might need adjuvant radiation therapy or androgen deprivation treatment.

Of note, the present findings have following limitations that merit mentioning. First and foremost, retrospective design is the primary limitation, which only allowed us to evaluate the temporal link between BCR and MetS, thereby causal inference is restricted, and the data we analyzed retrospectively, carries a potential selection bias for this study. Second, comorbidities of enrolled patients may not be precisely reported in the electronic medical record. Take an example, we were unable to make clear patients who didn't report the history of diabetes or hypertension to their medical providers. In this respect, we depended on self-reported information, leading to the possibility of misclassification bias. Also, some important information, such as diet, and physical activity, family history, and drug usage, including statins anti-diabetes drugs, was not collected. These latent confounders could limit the statistical power of the study. Third, the evaluation of obesity was carried out only by BMI in our study, rather than waist circumstance to define overweight, which might cause misclassification. Fourth, this study was a single-center study only in China, and the number of subjects was a small volume, which may not apply to all centers. Fifth, information pertaining to the duration of MetS and its components, which may theoretically influence BCR, was also deficient. Finally, the data of collected were fully based on Chinese men and thereby the results would not be generalized to other ethnic populations.

## Conclusion

In conclusion, the presence of BCR was associated with an increased risk of higher GS and positive nerve invasion. MetS and its components were not associated with BCR among the cohort of postoperative patients of our hospital in China. Prospective study using large multicentric patient groups with a long follow-up are warranted to more accurate verify the possible implication of MetS prevention on PCa recurrence.

## Data Availability Statement

The datasets generated for this study are available on request to the corresponding author.

## Ethics Statement

The studies involving human participants were reviewed and approved by First Affiliated Hospital of Zhejiang University. Written informed consent for participation was not required for this study in accordance with the national legislation and the institutional requirements.

## Author Contributions

XX and XW contributed to the conception or design of the work. QL, CC, and LX contributed to the acquisition, analysis, or interpretation of data for the work. XX drafted the manuscript. XW and QL critically revised the manuscript. All authors gave final approval and agree to be accountable for all aspects of work ensuring integrity and accuracy.

### Conflict of Interest

The authors declare that the research was conducted in the absence of any commercial or financial relationships that could be construed as a potential conflict of interest.
